# Neck strength alone does not mitigate adverse associations of soccer heading with cognitive performance in adult amateur players

**DOI:** 10.1371/journal.pone.0302463

**Published:** 2024-05-16

**Authors:** Chin Lun Lin, Bluyé DeMessie, Kenny Ye, Shanshan Hu, Michael L. Lipton

**Affiliations:** 1 Montefiore Medical Center, Bronx, New York, United States of America; 2 Department of Neuroscience, Albert Einstein College of Medicine, Bronx, New York, United States of America; 3 Department of Epidemiology and Population Health, Albert Einstein College of Medicine, Bronx, New York, United States of America; 4 Department of Systems and Computational Biology, Albert Einstein College of Medicine, Bronx, New York, United States of America; 5 The Epoch Times, New York, New York, United States of America; 6 Department of Radiology, Columbia University Irving Medical Center, New York, New York, United States of America; 7 Department of Biomedical Engineering, Columbia University, New York, New York, United States of America; Portugal Football School, Portuguese Football Federation, PORTUGAL

## Abstract

**Objectives:**

Soccer heading is adversely associated with neurocognitive performance, but whether greater neck strength or anthropometrics mitigates these outcomes is controversial. Here, we examine the effect of neck strength or anthropometrics on associations of soccer heading with neurocognitive outcomes in a large cohort of adult amateur players.

**Methods:**

380 adult amateur league soccer players underwent standardized measurement of neck strength (forward flexion, extension, left lateral flexion, right lateral flexion) and head/neck anthropometric measures (head circumference, neck length, neck circumference and neck volume). Participants were assessed for heading (HeadCount) and cognitive performance (Cogstate) on up to 7 visits over a period of two years. Principal components analysis (PCA) was performed on 8 neck strength and anthropometric measures. We used generalized estimating equations to test the moderation effect of each of the three PCs on 8 previously identified adverse associations of 2-week and 12-month heading estimates with cognitive performance (psychomotor speed, immediate verbal recall, verbal episodic memory, attention, working memory) and of unintentional head impacts on moderate to severe central nervous system symptoms.

**Results:**

3 principal components (PC’s) account for 80% of the variance in the PCA. In men, PC1 represents head/neck anthropometric measures, PC2 represents neck strength measures, and PC3 represents the flexor/extensor (F/E) ratio. In women, PC1 represents neck strength, PC2 represents anthropometrics, and PC3 represents the F/E ratio. Of the 48 moderation effects tested, only one showed statistical significance after Bonferroni correction, which was not robust to extensive sensitivity analyses.

**Conclusion:**

Neither neck strength nor anthropometrics mitigate adverse associations of soccer heading with cognitive performance in adult amateur players.

## Introduction

Soccer is the most widely played sport worldwide, with more than 265 million currently active players [1]. Head impacts are common in soccer and include player-to-player, player-to-goalpost and player-to-ground collisions (also known as unintentional head impacts) as well as “heading” where players intentionally use their unprotected head to deliberately direct the ball in play [2]. In addition to concussion, soccer players are at risk for potentially adverse effects of these repetitive head impacts (RHI). Multiple prior studies have shown that RHI is associated with adverse structural and functional brain effects, including lower white matter integrity, lower scores on neuropsychological testing and greater psychological distress [3–15]. These effects appear to be independent of the effects of unintentional head impacts and concussion [16, 17].

One of the main functions of the neck musculature is to provide stability for the head. A soccer player’s head velocity and acceleration during heading is limited by the contraction of neck musculature in reaction the impulse caused by contact of the ball with the head [18]. However, the more than 25 neck muscle pairs have complex lines of action and multiple points of insertion which make it challenging to study neck biomechanics [19]. The extent to which the neck resists change in position when a force is applied, is determined by active contraction of neck musculature and the passive resistance to compression by tendons, ligaments, and bone structure. Appropriate neuromuscular control of the relevant neck muscles is necessary to stabilize the head in the response to an impact [20, 21]. Neural control of neck musculature is dictated, in part, by the biomechanical constraints intrinsic to the individual [19].

Neck strength and anthropometrics control head and neck kinematics and may incorporate the torso as an effective mass [12]. The resulting strain on the neck and head can lead to a range of injuries following sport-related head impacts [22]. A study of youth and collegiate soccer players (n = 100) found that athletes with smaller head and neck size and lower neck strength were at risk for greater head acceleration [12]. Neck strength is hypothesized to be protective against head and neck injuries because stronger necks would better stabilize the head during an impact [23–25]. Studies on the protective role of greater neck strength in sports have shown mixed results. Neck strength was not found to be predictive of concussion risk for ice hockey players [26]. However, a small study (n = 17) of adolescent female soccer players found that greater neck strength is negatively correlated with head acceleration during heading [9], and a large study (n = 6,704) of male and female adolescent basketball, soccer, and lacrosse athletes found that for every 1 pound increase in neck strength, the odds of concussion decreased by 5% [27]. A recent systematic review by Elliott et al. found that higher neck strength may be associated with a lower risk of sport-related concussion [24]. While these latter studies identified a potential role for neck strength in prevention of recognized concussion, they did not address the role of RHI, which entail many impacts over time that do not lead to recognized concussion.

Understanding the potential for neck strength to mitigate adverse effects of RHI in sport could lead to identification of a modifiable risk factor [28, 29] for the previously described adverse effects of sports-related RHI [3, 30–32]. In addition, RHI from sports have been proposed to be a potential mechanism leading to the development of neurocognitive decline later in life [33, 34]. If neck strength has the potential to reduce the risk for adverse consequences of soccer heading, neck strengthening exercises could be a protective measure to reduce head acceleration and risk of adverse effects from RHI.

The purpose of this study was to test the hypothesis that neck strength or anthropometrics moderate previously reported adverse associations of both short-term (2 weeks) and long-term (12 months) soccer RHI exposure with neurocognitive performance, and with central nervous system (CNS) symptoms in adult amateur players [3, 30–32]. Based on the literature [8, 12], we hypothesize that neck strength (forward flexion, extension, left lateral flexion, right lateral flexion) and anthropometrics (head circumference, neck length, neck circumference and neck volume) moderate adverse associations of soccer RHI exposure with CNS symptoms.

## Methods

The Einstein Soccer Study is a multifaceted longitudinal study of repetitive head impacts (RHI) and its consequences in adult amateur soccer players [30]. We analyzed prospectively collected data including assessments of RHI exposure, cognitive performance, and neck strength and anthropometrics. The same data collection and assessments were used for all participants.

This study was carried out in accordance with the recommendations of the Albert Einstein College of Medicine Institutional Review Board with written informed consent from all subjects.

Statistical power was based on published and preliminary data on the association of RHI with cognitive outcomes used in the Einstein Soccer Study cohort [35]. We utilized all available participant data without exclusion to minimize bias. Participants were 380 adult amateur soccer players who completed a total of 1,307 study visits (1–7 visits per participant over a two-year period in a longitudinal study of soccer play and its consequences). Soccer players were recruited between November 2013 and May 2018 by print and Internet advertisement and through soccer leagues and clubs in New York City and surrounding areas. Interested individuals were directed to an enrollment website, which, after informed consent, collected screening information. A research team member contacted qualifying individuals, confirmed eligibility, willingness to participate in the study and invited to enroll. Inclusion criteria were: aged 18–55 years old; at least 5 years of active amateur soccer play; current active amateur soccer play; at least 6 months of amateur soccer play annually; and English language fluency. Participants were asked to report neurological or other medical diagnoses. Exclusion criteria were: schizophrenia, bipolar disorder; current neurological disorder; pregnancy; and medical contraindication to MRI. Details of the overall study design have been published previously [17, 35].

### Heading assessment

We used HeadCount, a suite of web-based assessments that estimate heading and unintentional head impacts (e.g., collision with another player) over distinct timeframes as well as lifetime concussions. HeadCount-2w provides estimates of exposure over the prior 2 weeks and HeadCount-12m over the prior 12 months. Prior publications have reported on HeadCount in detail [36, 37]. Briefly, participants were asked questions relative to their soccer activity in indoor and outdoor settings including, as appropriate to the timeframe (2 weeks or 12 months), the number of months played per year, the mean number of competitive soccer games per week, the mean number of headers per game, the mean number of practices per week, and the mean number of headers per practice. Total number of headers in the past two weeks was estimated by multiplying the number of headers in each setting (indoor or outdoor) by the number of number of sessions (practice or game) per week for each setting and multiplying each result by two. Total two-week exposure was estimated as the sum of the four resulting subtotals (indoor/outdoor x practice/game). The total number of headers in the past year was estimated by multiplying the mean number of headers in each setting (indoor or outdoor) by the number of sessions (practice or game) per week in each setting, converting to months, and then multiplying by the number of months of play per year. Subtotals for each setting were summed to obtain an estimate of total 12-month heading.

Due to the high degree of right skew in the exposure measures, we treated each as a categorical variable. Heading was treated as an ordered categorical variable of approximately equal size quartiles across both men and women. Quartile ranges for headers in the past two weeks were: Q1: 0 (no heading), Q2: 1–8 headers, Q3: 9–28 headers, Q4: more than 28 headers. For heading over the past year, quartiles ranges were: Q1: 0–290 headers, Q2: 291–678 headers, Q3: 679–1781 headers and Q4: more than 1,781 headers.

In addition to heading, participants were asked how often while playing soccer in the past 2 weeks they experienced unintentional head impacts, such as a player’s head hitting another player’s head, elbow, knee, the ground or being hit by the ball in the back of the head. The number of unintentional impacts over the past 2 weeks were categorized as “zero”, “1” and “2 or more”.

### Neuropsychological assessment

We tested the modifying effect of neck strength on previously reported adverse relationships [17, 35] of short and long term head impact exposure with cognitive performance measures using the Cogstate test battery (Cogstate Ltd., NY, USA) [38] at each visit. We focused our moderation analyses on the following previously reported adverse associations: (1) 2-week heading associated with performance on the Groton Maze Chase Task (GMCT), which measures psychomotor speed by assessing how quickly and accurately participants chase a target through a maze; (2) 2-week and (3) 12-month heading associated with performance on the International Shopping List Task, immediate recall (ISL), which measures verbal learning; (4) 2-week and (5) 12-month heading associated with performance on the International Shopping List Task, 20-minute delayed recall (ISLR), which measures verbal episodic memory; (6) 2-week heading associated with performance on the One Back Task (ONB), which assesses attention; (7) 2-week heading associated with performance on the Two Back Task (TWOB), which measures working memory. In addition, we tested the modifying effect of neck strength on our previous finding that (8) unintentional head impacts are associated with moderate to severe CNS symptoms in the past 2 weeks [30]. Moderate CNS symptoms were moderate pain or some dizziness and severe CNS symptoms were feeling dazed, needing to stop playing or requiring medical attention [30].

### Neck strength and anthropometrics testing

In this study, 4 neck strength measures and 4 anthropometric measures were obtained using previously described methods including fixed-frame dynamometry [39]. To determine the reliability of our method of FFD for measuring neck strength, 10 subjects (5 male and 5 female) participated in an additional 4 testing sessions over a 2-week period. Each testing session occurred on a different day. To account for variability attributable to subject positioning and/or verbal coaching, for each subject, 2 different raters alternated across the 4 visits. The sequence of raters was counterbalanced across subjects.

#### Neck strength

At each baseline and follow-up visit, 3 trials of isometric force were measured for four separate conditions (forward flexion, extension, left lateral flexion, right lateral flexion) by using fixed-frame dynamometry technique with a MicroFet2 digital dynamometer (Hoggan Scientific, Salt Lake City, UT) as described in Catenaccio et al [39]. The dynamometer has a test range of 12.1 Newtons (N) to 1320N in 0.4N increments [40]. Neck strength was measured, as detailed below, in 4 directions: forward flexion, extension, left lateral flexion, right lateral flexion. The testing order for the 4 directions was randomized using the randomization feature in RedCap [41].

The peak force trials for each condition across three trials were used in our analyses. We also calculated the ratio of forward flexion to extension as an estimate of neck strength imbalance, as commonly reported in the literature [25, 42–44].

During testing sessions, subjects were seated in a custom-built rigid chair with 2 seatbelts attached just below the axillae and at the waist. To minimize contributions from thoracic and abdominal musculature, seatbelts were tightened until subjects were unable to separate their torso from the back of the chair. To minimize contributions from the scapular and pectoral muscles and to prevent bracing against the chair, subjects’ arms were positioned with 90° abduction at the shoulders and 90° flexion at the elbows [45]. To minimize contributions of lower extremity muscles, the subject was asked to rest the feet lightly on top of an empty cardboard box and instructed not to exert any pressure on the box during testing [46]. Trials in which the box was crushed by the subject were discarded and repeated. The position of the dynamometer was adjusted for each direction: on the occipital protuberance for extension, above the brow ridge in the midline for forward flexion, and above the corresponding ear for right lateral flexion and left lateral flexion.

Subjects were encouraged, with verbal coaching, to push against the dynamometer continuously with full effort for 3–4 seconds during each trial. Three trials were completed for each direction with a 5-second rest between trials and a 30-second rest between directions. The dynamometer recorded peak force for each 3-second trial in Newtons (N).

#### Anthropometric measures

Anthropometric measures included head circumference, neck length, neck circumference and neck volume (defined as the product of neck length and the square of neck circumference divided by 4π), measured at each study visit. Neck circumference was taken immediately cranial to the thyroid cartilage, with the head in a neutral position. Neck length was measured from the most prominent vertebral spinous process (the seventh cervical vertebra) to the occipital protuberance, with the subject’s chin relaxed toward the chest and the tape measure flush against the curve of the neck. Head circumference was measured at the brow ridge and occipital protuberance.

### Statistical analysis

The motivation for our statistical approach was to reduce the dimensionality of neck isometric strength and anthropometrics by principal component analysis and test the moderating effects using only the top principal components. This approach serves two purposes, mitigating multiple testing, and avoiding collinearity in the regression models. For principal component analysis, each of the 8 neck strength and anthropometric measures was averaged over all visits for each player. Principal component analysis on the 8 variables was performed using the function principal of the R library psych 2.3.6) [47] that applies a varimax rotation [48–50] to the top three principal components to make them more interpretable. These PCs were then tested as potential modifiers of the associations of heading with cognitive performance.

We employed generalized estimating equations (GEE; geepack in R 4.2.2) [51, 52] to account for correlations among cognitive outcomes measured at multiple visits, with an exchangeable correlation structure for the error terms. The corresponding heading exposure (i.e., quartile) and a neck-strength or anthropometric principal component were the explanatory variable of interests. Age, years of education, alcohol use (none, light, moderate/heavy), years of soccer play and lifetime concussion history (zero, one, two or more) were included in all models as covariates.

The statistical significance of the moderating effect was obtained using the Wald ANOVA test on the overall interaction effect between each neck principal component and the heading exposure variable. Since we have a total of 48 tests, the significance level is set at α = 0.001 based on the Bonferroni correction, so that the overall Type-I error is below 0.05.

## Results

### Participant characteristics and sex differences

**Fig 1:**
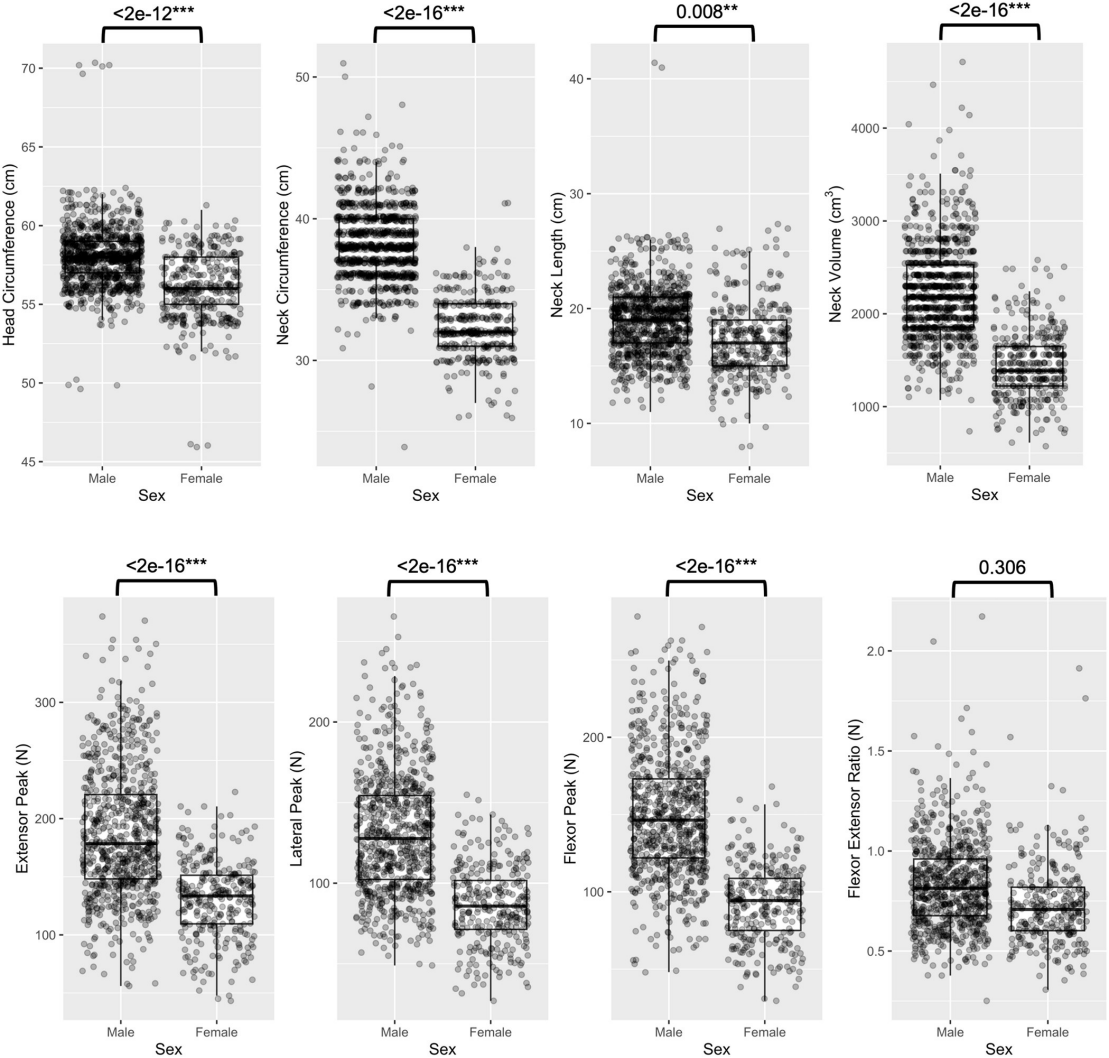
Neck strength and anthropometric measures for men and women. Welch Two Sample t-test: *p<0.05; **p<0.01; ***p<0.001.

Participant characteristics at baseline are shown in Table 1. Women and men differed greatly on nearly all anthropometric and strength measures, with women exhibiting lesser neck strength compared to males (Fig 1). To avoid confounding, subsequent analyses were stratified by sex.

**Table 1:** Participant characteristics.

Variable	Male, n = 266^1^	Female, n = 114^1^
**Age (y)**	26 (7)	26 (8)
**Height (cm)**	176 (7)	163 (8)
**Body Mass (kg)**	77 (11)	63 (9)
**BMI**	25.0 (3.2)	23.7 (3.4)
**% Right-Handed**	0.75 (0.45)	0.86 (0.25)
**Years Played**	12 (7)	12 (8)
**Years Education**	16 (2)	16 (2)
**Smoking**	82 (32%)	25 (24%)
**Alcohol Use**		
I do not drink	66 (26%)	23 (22%)
1–2 drinks per week	108 (42%)	44 (43%)
3–7 drinks per week	61 (24%)	33 (32%)
8–14+ drinks per week	23 (8.9%)	3 (2.9%)
**Main Soccer Position**		
Forward	55 (21%)	17 (15%)
Midfield	92 (35%)	44 (40%)
Defense	99 (38%)	39 (35%)
Goaltender	18 (6.8%)	11 (9.9%)
**Concussion History**		
0	186 (70%)	56 (50%)
1	38 (14%)	22 (19%)
2+	41 (15%)	35 (31%)

^1^. Mean (SD) or n (%)

### Characterization of modifier variables

Principal Component Analysis (PCA) on the 8 neck strength and anthropometric variables showed that the 3 top principal components together explain about 80% of the total variation, for both men and women. These PCs were used in subsequent analyses.

In males, the first principal component (PC1) represents primarily head/neck anthropometric measures, with the second principal component (PC2) representing neck strength measures and the third (PC3) representing the flexor/extensor (F/E) ratio. In females, PC1 represents neck strength, with PC2 representing anthropometrics and PC3 the F/E ratio (S1 Table).

### Tests of moderation by neck strength and anthropometrics

Results are shown in Table 2 for moderation by each of the three principal components of eight previously reported adverse associations, 7 of short- or long-term heading with measures of cognitive performance and one of unintentional head impacts with CNS symptoms (see Methods). Out of 48 tests, only PC1 for women modified the effect of 12mo-heading on ISL at a significance level that exceeded the Bonferroni-corrected significance threshold. The details for this regression model are shown in S2 Table.

**Table 2:** Moderating effect of neck strength or anthropometrics on associations of heading with outcome measures.

			Male	Female
Outcome^1^	Predictor	Moderator^2^	p-value^3^	p-value^3^
GMCT	2wk-heading	PC1	0.70	0.41
		PC2	0.08	0.90
		PC3	0.11	0.36
ISL	12mo-heading	PC1	0.04	8e-04*
		PC2	0.84	0.34
		PC3	0.66	0.14
ISL	2wk-heading	PC1	0.47	0.74
		PC2	0.82	0.19
		PC3	0.41	0.36
ISRL	12mo-heading	PC1	0.58	0.01
		PC2	0.73	0.34
		PC3	0.69	0.13
ISRL	2wk-heading	PC1	0.37	0.12
		PC2	0.31	0.07
		PC3	0.75	0.65
Symptoms	Unintentional head impacts	PC1	0.96	0.61
		PC2	0.89	1e-03
		PC3	0.87	0.86
ONB	2wk-heading	PC1	0.41	0.38
		PC2	0.72	0.77
		PC3	0.86	0.07
TWOB	2wk-heading	PC1	0.36	0.27
		PC2	0.93	0.95
		PC3	0.82	0.02

^1^. GMCT = Groton Maze Chase Task, ISL = International Shopping List—immediate recall, ISRL = International Shopping List—delayed recall, Symp = CNS symptoms past 2 weeks, ONB = One Back Task, TWOB = Two Back Task

^2^. PC = Principal component (representing neck strength or anthropometrics), see S1 Table for more details

^3^. ANOVA test of overall interaction effect: *p<0.001 (Bonferroni significance cutoff)

Sensitivity analyses (S4 and S5 Tables) showed that exclusion of participants over 50 years did not alter the results, from which we infer that inclusion of these older individuals, who were part of the study cohort, did not bias the regression models.

## Discussion

The purpose of this study was to investigate whether neck strength or anthropometrics moderate adverse associations of soccer heading or unintentional head impacts with cognitive performance or CNS symptoms among adult amateur players. Among male soccer players, neither neck strength nor anthropometrics modified associations of either soccer heading or unintentional head impacts with either cognitive performance or CNS symptoms. Among female soccer players, PC1 (representing neck strength) moderated the association of 12 month heading with ISL (Table 2). The direction of this association suggests that greater neck strength worsens the adverse association of heading with cognitive performance among female soccer players. No other moderating effect of either neck strength or anthropometrics with either cognitive performance or CNS symptoms met the Bonferroni-corrected significance threshold.

Prior studies have reported potential mechanisms that could explain the unique moderating effect we identified for women. Potential ways in which our findings could be understood in light of prior results include: (a) prior research has found women are more vulnerable to adverse effects of soccer heading on cognitive performance [53] and white matter microstructure [54]; (b) women with greater neck strength might perform heading drills during practice which have been associated with adverse effects [55]; (c) greater neck strength could reflect a style of play that alters risk, such as heading while standing vs jumping [56]; (d) suboptimal heading technique could be more prevalent among female players with greater neck strength, altering risk [57, 58]; or (e) greater neck strength among women might confer imbalanced neck stabilization, which has been found to lead to greater angular head acceleration during heading among collegiate players [59].

However, the statistical significance of this modifying effect was not robust to various sensitivity analyses we conducted, which included: (1) combined analysis of male and female players to increase sample size; (2) including both peak and average neck strength in the PCA; (3) imputing missing values; and (4) using an independent vs exchangeable correlation structure in the GEE.

In addition to the potential mechanistic explanations (a-e, above), we also note that since the sample size for female players is smaller than for men, this unique finding could be spurious and warrants replication in larger samples. We therefore conclude that our findings do not support mitigation by isometric neck strength alone of previously identified adverse associations of soccer heading with cognitive performance among either men or women.

Although stronger neck musculature may indeed stabilize the head and reduce head acceleration, the notion that strength alone mitigates deleterious cognitive effects of heading is not supported by our results. We also found that neck strength did not modify associations of unintentional head impacts with CNS symptoms. Although our findings in adult amateurs contrast with prior studies in pediatric cohorts, which suggested neck strength would protect against concussion from unintentional impacts [9, 27], these prior results may not be generalizable to adults who have larger head mass, increased neck girth/strength and more developed heading skills [60]. Additionally, neck strength may only confer benefit when muscle activation occurs at the time of impact [9, 14, 15, 61, 62], which is less likely to occur during an unanticipated impact.

In addition to our primary assessment of direct modifying effects of neck strength or anthropometrics, we explored two other areas related to neck strength among soccer players. First, we found, in line with previous studies, that anthropometrics are poor predictors of neck strength (S3 Table) [27, 39], suggesting that anthropometrics cannot serve as effective surrogates for direct neck strength measures. Second, we found heading to show little association with neck strength in both men and women, suggesting that high frequency of heading may not improve neck strength and that players with stronger necks may not necessarily head more often (S6 Table). As we did not find a modifying effect of isometric neck strength, these findings may seem insignificant. However, it has been shown in professional soccer players that higher heading exposure was associated with lower severity of symptoms of concussion, depression, anxiety, and sleep disorders [63]. While neck strength alone does not appear to modify the association of heading with cognitive performance, it may still moderate the effect of heading on subclinical structural brain features such as white matter microstructure [54] and could have relevance when viewed in the context of timing muscle activation to the moment of impact [9, 14, 15, 61, 62]. Indeed, without testing other aspects of neck function (e.g., timing of neck muscle contraction), we cannot be sure if the lack of a moderating effect by neck strength observed in our research is limited to the role of static isometric neck strength or could indicate that neck function overall does not mitigate effects of RHI. These remain important areas for future investigation.

Many prior studies supporting the role of neck strength in mitigation of adverse effects of RHI are limited by relatively small sample sizes (often fewer than 30 participants) [9, 64], limited ecological validity of the laboratory paradigms employed [20] or lack of control for relevant factors such as previous concussion [27]. Moreover, most of these studies reported on head acceleration, not functional outcomes, such as incidence of concussion, symptoms, or cognitive/structural effects [11, 65]. Thus, it remains an open question whether the biomechanical effect of neck strength on head acceleration, demonstrated in prior studies [8, 9, 65, 66], prevents injury or dysfunction [24, 62]. Our study addresses many of these gaps in knowledge, with a large sample size, observational design assessing real-world soccer play and cognitive performance, and statistical modeling that accounts for potential confounders.

Confirmation that neck strength stabilizes the head during sport-related head impacts and reduces the risk of brain injury in the context of soccer RHI, could set the stage for the use of neck strengthening as a protective feature in soccer [23, 66]. Mansell et al. found that neck strength training in collegiate male and female soccer players did not affect head acceleration during head impacts [14]. Research into neck strength training in managing the risk to concussion suggests that neck stiffness at the time of impact, rather than neck strength, may reduce the risk of concussion [29, 62, 67, 68]. Some have found that incorporating neck exercises results in a decrease in head acceleration by improving neuromuscular control [66, 69]. Whereas other have demonstrated that there is no reduction of head acceleration after isotonic neck exercises [70, 71]. Although there is limited literature on neck training to reduce head injury risk [72–74] or neurological changes [7, 75], no study to date has directly examined the effect of neck strength on the adverse effects of RHI, including heading.

The biomechanics of heading and head kinematics following impact are complex and are likely to vary not only due to neck strength but also with the timing of cervical muscle activation [20], and with heading technique, such as head-torso alignment and follow-through [60]. The benefits of neck strength might only be relevant when timing of muscle activation is synchronous with the moment of head impact [8, 24, 62]. Further research is warranted to determine if integration of strengthening with optimized aspects of heading technique could realize a significant protective effect.

## Limitations

Our findings must be considered in light of several limitations. Fixed-frame dynamometry is an established method for measurement of neck strength. However, it largely reflects isometric strength, which might not fully reflect the true biomechanics of head impact. Some papers have argued for studying muscle strength under dynamic conditions (e.g., isokinetic) using EMG, hypothesizing that the pattern and timing of muscle activation may be more important than absolute strength [29]. It is unclear whether neck strength imbalance, which could potentially worsen heading-related effects [10], is better assessed by the difference or ratio of flexor and extensor muscle strength. We chose to calculate the ratio of forward flexion to extension as an estimate of neck strength imbalance, which is the approach commonly reported in the literature [25, 42–44]. We performed a sensitivity analysis, which showed the two approaches made minimal differences in our model outcomes and did not alter significance. Our study might not have been sufficiently powered to detect a moderation effect, leading to false negative inferences. However, we studied a larger sample (380 participants, 266 men and 114 women, who completed a total of 1307 visits) than prior studies, which did identify significant adverse associations of heading with cognitive performance [5, 6, 30, 35]. Thus, we expect to be well-positioned to detect a moderation effect of next strength on these associations. The heading exposure variable we employ is derived from self-report, which may be subject to bias. However, the instruments we employ have been extensively validated [36, 37] and elicit similar exposure-response relationships across independent samples of players. The cognitive effects we attribute to heading could in part be due to unintentional head impacts. However, we previously reported in this cohort that heading, not unintentional impacts, explains variance in cognitive performance measures [17]. To confirm this, we performed a sensitivity analysis including unintentional impacts as a covariate, which did not change our results. One possibility is that unintentional impacts are comparably rare (median four per year), compared to heading (median 678 per year) in our sample, which may be insufficient exposure to affect cognitive performance. Finally, our results cannot explicitly be generalized beyond the adult amateur league players in this study. Other groups, especially collegiate or professional players could respond differently if they have much greater neck strength.

## Conclusion

Although prior research proposed isometric neck strength as a modifiable risk factor for brain injury due to soccer heading, our findings suggest that neither isometric neck strength nor head/neck anthropometric measures mitigate previously reported adverse associations of soccer heading with cognitive performance in adult amateur players. In addition, neither neck strength nor anthropometric measures mitigated previously reported associations of unintentional head impacts with CNS symptoms. Nonetheless, a more nuanced approach, which considers neck strength along with timing of muscle activation and optimal technique, might be necessary to identify the factors that can confer risk reduction. Future research should examine whether muscle strength under dynamic conditions moderates the association of repetitive head impacts with cognitive performance, CNS symptoms, and subclinical structural brain features of injury such as white matter microstructure.

## Supporting information

S1 Table Variable contributions (loadings) for the first 3 principal components. (DOCX)

S2 Table Regression model testing the modifying effect of PC1 on the association of 12 month heading with ISL among female soccer players. (DOCX)

S3 Table Proportion of neck strength variation explained by anthropometric measures. (DOCX)

S4 Table Moderating effect of neck strength on heading in participants younger than 50 years old. (DOCX)

S5 Table Regression model testing the modifying effect of PC1 on the association of 12 month heading with ISL among female soccer players younger than 50 years old. (DOCX)

S6 Table Spearman correlation of neck strength measures and heading. (DOCX)
