# Supplementary material for: Neck strength alone does not mitigate adverse associations of soccer heading with cognitive performance in adult amateur players
Source: PLoS One. 2024 May 16;19(5):e0302463. doi: 10.1371/journal.pone.0302463 (PMC11098408; doi:10.1371/journal.pone.0302463)
Supplement: S1 Table — (DOCX) [file pone.0302463.s001.docx]

Table S1. Variable contributions (loadings) for the first 3 principal components

|  | **Male** | | | **Female** | | |
| --- | --- | --- | --- | --- | --- | --- |
| **Variable** | **PC1** | **PC2** | **PC3** | **PC1** | **PC2** | **PC3** |
| **Neck strength measures** | | | | | | |
| Extension Peak (N) | 0.02 | 0.71 | -0.66 | 0.88 | 0.08 | -0.36 |
| Flexion Peak (N) | 0.10 | 0.92 | 0.18 | 0.79 | 0.11 | 0.54 |
| Lateral Peak (N) | 0.15 | 0.85 | -0.03 | 0.89 | 0.09 | 0.01 |
| F/E Ratio^1^ | 0.08 | 0.12 | 0.96 | -0.09 | 0.04 | 0.98 |
| **Anthropometric measures** | | | | | | |
| Neck Circ (cm) | 0.66 | 0.35 | 0.20 | 0.39 | 0.58 | -0.04 |
| Head Circ (cm) | 0.49 | 0.15 | 0.04 | 0.05 | 0.43 | 0.17 |
| Neck Length (cm) | 0.82 | -0.18 | -0.11 | -0.08 | 0.87 | -0.04 |
| Neck Volume (cm3) | 0.97 | 0.10 | 0.05 | 0.14 | 0.97 | -0.05 |
| ^1^Defined as Flexion / Extension | | | | | | |
