# Supplementary material for: Neck strength alone does not mitigate adverse associations of soccer heading with cognitive performance in adult amateur players
Source: PLoS One. 2024 May 16;19(5):e0302463. doi: 10.1371/journal.pone.0302463 (PMC11098408; doi:10.1371/journal.pone.0302463)
Supplement: S2 Table — (DOCX) [file pone.0302463.s002.docx]

Table S2. Regression model testing the modifying effect of PC1 on the association of 12 month heading with ISL among female soccer players

| **Variable** | **Beta** | **95% CI^1^** | **p-value** |
| --- | --- | --- | --- |
| **1yr-Heading** |  |  |  |
| 0-289.25 | — | — |  |
| 289.26-678 | -0.59 | -1.9, 0.76 | 0.4 |
| 679-1781 | -1.9 | -3.7, -0.16 | 0.033 |
| 1782+ | -0.96 | -2.5, 0.58 | 0.2 |
| **PC1** | 0.56 | 0.04, 1.1 | 0.036 |
| **1yr-Heading * PC1** |  |  |  |
| 289.26-678 * PC1 | -1.7 | -2.9, -0.58 | 0.003 |
| 679-1781 * PC1 | 1.3 | -0.34, 2.9 | 0.12 |
| 1782+ * PC1 | -0.94 | -2.0, 0.11 | 0.078 |
| ^1^CI = Confidence Interval | | | |
| n = 114 female soccer players, 367 total visits. Outcome: International shopping list - immediate recall (ISL). | | | |
