# Supplementary material for: Neck strength alone does not mitigate adverse associations of soccer heading with cognitive performance in adult amateur players
Source: PLoS One. 2024 May 16;19(5):e0302463. doi: 10.1371/journal.pone.0302463 (PMC11098408; doi:10.1371/journal.pone.0302463)
Supplement: S3 Table — (DOCX) [file pone.0302463.s003.docx]

Table S3. Proportion of neck strength variation explained by anthropometric measures

| **Outcome** | **Male^1^** | **Female^1^** |
| --- | --- | --- |
| Extension Peak (N) | 0.07 | 0.13 |
| Flexion Peak (N) | 0.18 | 0.12 |
| Lateral Peak (N) | 0.16 | 0.12 |
| ^1^r-squared: proportion of variance explained by a linear model of anthropometric variables, including age, height, weight, neck length, neck circumference and head circumference. | | |
