# Supplementary material for: Neck strength alone does not mitigate adverse associations of soccer heading with cognitive performance in adult amateur players
Source: PLoS One. 2024 May 16;19(5):e0302463. doi: 10.1371/journal.pone.0302463 (PMC11098408; doi:10.1371/journal.pone.0302463)
Supplement: S4 Table — (DOCX) [file pone.0302463.s004.docx]

Table S4. Moderating effect of neck strength on heading in participants younger than 50 years old.

|  | | | **Male** | **Female** |
| --- | --- | --- | --- | --- |
| **Outcome^1^** | **Predictor** | **Moderator^2^** | **p-value^3^** | **p-value^3^** |
| GMCT | 2wk-heading | PC1 | 0.71 | 0.58 |
| GMCT | 2wk-heading | PC2 | 0.18 | 0.96 |
| GMCT | 2wk-heading | PC3 | 0.63 | 0.25 |
| ISL | 12mo-heading | PC1 | 0.03 | 7e-06* |
| ISL | 12mo-heading | PC2 | 0.66 | 0.36 |
| ISL | 12mo-heading | PC3 | 0.35 | 0.09 |
| ISL | 2wk-heading | PC1 | 0.13 | 0.99 |
| ISL | 2wk-heading | PC2 | 0.02 | 0.13 |
| ISL | 2wk-heading | PC3 | 0.82 | 0.30 |
| ISRL | 12mo-heading | PC1 | 0.38 | 1e-03 |
| ISRL | 12mo-heading | PC2 | 0.45 | 0.32 |
| ISRL | 12mo-heading | PC3 | 0.60 | 0.35 |
| ISRL | 2wk-heading | PC1 | 0.74 | 0.04 |
| ISRL | 2wk-heading | PC2 | 0.35 | 0.36 |
| ISRL | 2wk-heading | PC3 | 0.64 | 0.55 |
| Symp | Unint-impacts | PC1 | 0.98 | 0.57 |
| Symp | Unint-impacts | PC2 | 0.85 | 3e-03 |
| Symp | Unint-impacts | PC3 | 0.90 | 0.83 |
| ONB | 2wk-heading | PC1 | 0.56 | 0.19 |
| ONB | 2wk-heading | PC2 | 0.99 | 0.83 |
| ONB | 2wk-heading | PC3 | 0.97 | 0.14 |
| TWOB | 2wk-heading | PC1 | 0.47 | 0.38 |
| TWOB | 2wk-heading | PC2 | 0.67 | 0.93 |
| TWOB | 2wk-heading | PC3 | 0.63 | 0.02 |
| ^1^GMCT = Groton Maze Chase Task, ISL = International Shopping List - immediate recall, ISRL = International Shopping List - delayed recall, Symp = CNS symptoms past 2 weeks, ONB = One Back Task, TWOB = Two Back Task | | | | |
| ^2^PC = Principal component (representing neck strength or anthropometrics), see Supplemental Table 4 for more details | | | | |
| ^3^ANOVA test of overall interaction effect: *p<0.001 (Bonferroni cutoff) | | | | |
