# Supplementary material for: Neck strength alone does not mitigate adverse associations of soccer heading with cognitive performance in adult amateur players
Source: PLoS One. 2024 May 16;19(5):e0302463. doi: 10.1371/journal.pone.0302463 (PMC11098408; doi:10.1371/journal.pone.0302463)
Supplement: S5 Table — (DOCX) [file pone.0302463.s005.docx]

Table S5. Regression model testing the modifying effect of PC1 on the association of 12 month heading with ISL among female soccer players younger than 50 years old.

| **Variable** | **Beta** | **95% CI^1^** | **p-value** |
| --- | --- | --- | --- |
| **1yr-Heading** |  |  |  |
| 0-289.25 | — | — |  |
| 289.26-678 | -0.59 | -1.9, 0.75 | 0.4 |
| 679-1781 | -2.1 | -3.7, -0.56 | 0.008 |
| 1782+ | -0.74 | -2.2, 0.75 | 0.3 |
| **PC1** | 0.95 | 0.40, 1.5 | <0.001 |
| **1yr-Heading * PC1** |  |  |  |
| 289.26-678 * PC1 | -2.2 | -3.4, -1.1 | <0.001 |
| 679-1781 * PC1 | 1.2 | -0.29, 2.8 | 0.11 |
| 1782+ * PC1 | -1.2 | -2.2, -0.23 | 0.016 |
| ^1^CI = Confidence Interval | | | |
| n = 111 female soccer players, 356 total visits. Outcome: International shopping list - immediate recall (ISL). | | | |
