# Supplementary material for: Neck strength alone does not mitigate adverse associations of soccer heading with cognitive performance in adult amateur players
Source: PLoS One. 2024 May 16;19(5):e0302463. doi: 10.1371/journal.pone.0302463 (PMC11098408; doi:10.1371/journal.pone.0302463)
Supplement: S6 Table — (DOCX) [file pone.0302463.s006.docx]

**Table S6. Spearman correlation of neck strength measures and heading**

| **Neck Strength** | **Heading** | **ρ** |
| --- | --- | --- |
| Flexor Peak | 2wk-heading | 0.171 |
| Flexor Peak | 12mo-heading | 0.196 |
| Extension Peak | 2wk-heading | 0.146 |
| Extension Peak | 12mo-heading | 0.123 |
| Lateral Peak | 2wk-heading | 0.162 |
| Lateral Peak | 12mo-heading | 0.187 |
